# Colorimetric method for rapid detection of Oxacillin resistance in *Staphylococcus aureus* and its comparison with PCR for *mec* A gene

**DOI:** 10.1038/srep23013

**Published:** 2016-03-10

**Authors:** Niraj Ghanwate, Prashant Thakare, P. R. Bhise, Sonali Gawande

**Affiliations:** 1 Department of Microbiology, Sant Gadge Baba Amravati University, Amravati, 444602, MS, India; 2 Department of Biotechnology, Sant Gadge Baba Amravati University, Amravati, 444602, MS, India; 3 Department of Microbiology, DR. P D M Medical College, Amravati, 444603, MS, India

## Abstract

Rapid and accurate detection of Methicillin Resistant *Staphylococcus aureus* (MRSA) is an important role of clinical microbiology laboratories to avoid treatment failure. The detection of MRSA is based on phenotypic assays which require at least 24 h to perform. Detection of the *mecA* gene or of PBP 2a is the “gold standard”, but not always available. The aim of this study was to evaluate a rapid method for detection of MRSA by using 3 (4, 5 dimethyl thiazole -2-yl) -2, 5 diphenyl tetrazolium bromide (MTT). Total 126 isolates of MRSA were collected from tertiary healthcare center and were confirmed by oxacillin screening agar test as per CLSI guidelines. Amplification of *mec*A gene was performed by using PCR. MTT assay was carried out for all the isolates in 96 well Microtitre plate and compared with standard methods of CLSI. Out of 126 isolates, 98 were found to be *mec*A positive. MTT method was found to be 98.98% sensitive and 96.43% specific. The MTT based colorimetric method is rapid and simple test for screening of oxacillin resistance in *Staphylococcus aureus*. It significantly shortens the time to just **7 h** required to obtained a drug susceptibility test and could be useful to screen MRSA.

Methicillin resistant *Staphylococcus aureus* (MRSA) strains emerged soon after the introduction of methicillin into clinical practice. MRSA is one of the major pathogen associated with serious nosocomial infection because this strain shows multiple drug resistance which limits treatment possibilities^1^. Numerous clinical studies have indicated, based on mortality rates, that MRSA strains are more virulent than methicillin-susceptible *S. aureus* (MSSA) strains^2^. The proportion of patients whose death is attributable to MRSA is significantly higher than that for MSSA^3^. Resistance to oxacillin is mostly mediated by the *mecA* gene, which codes for the production of a supplemental penicillin-binding protein, PBP2a or 2′, which is expressed either homogeneously or heterogeneously^4^,^5^. Expression of resistance in some MRSA strains is also regulated by homologues of the regulatory genes for *blaZ* that encodes for β-lactamase. These genes, *mecI* and *mecR1*, regulate the *mecA* response to β-lactam antibiotics in a fashion similar to that of the regulation of *blaZ* by the genes *blaR1* and *blaI* upon exposure to penicillin^6^. Rosato *et al.*^7^ have found that either *mecI* or *blaI* must be functional in all MRSA. An additional series of genes, the *fem* genes (factor essential for resistance to methicillin resistance), play a role in cross-linking peptidoglycan strands and also contributes to the heterogeneity of expression of methicillin resistance^8^. The typical heterogeneity seen in the expression of resistance to methicillin and in levels of resistance depends on the concerted action of chromosomally encoded genes, including fem and aux that are also present in the genome of susceptible staphylococci. Early detection of drug resistance is one of the essential steps in the management of MRSA infections and the effectiveness of a standard Anti-MRSA treatment regimen correlates well with the *in-vitro* drug susceptibility pattern of infecting methicillin resistant *Staphylococci*^5^. MRSA has been detected by Disk diffusion method, Agar dilution method and Oxacillin Screen agar test recommended by Clinical Laboratory Standard Institution. In number of clinical microbiology laboratories performing routine work for the detection of MRSA is based on phenotypic assay such as disk diffusion and broth micro dilution^9^. Culture requires longer turnaround time of about 18–48 h with low sensitivity and 100% specificity^10^. Whereas detection of *mec*A gene or PBP2a is the ‘Gold Standard’ offering high sensitivity and rapid results but these methods are not always been possible in many facilities constrains laboratories^11^ of developing countries like India. For this reason rapid and reliable antibiotic susceptibility testing method for detection of MRSA is needed^12^. Therefore the aim of our study was to evaluate the MTT based colorimetric method for rapid detection of Oxacillin Resistant *Staphylococcus aureus* and to compare it with the CLSI methods and PCR for *mec* A. MTT is a yellow tetrazolium salt which is converted into a blue formazan by dehydrogenase of live cell. This method is based on the principle that the amount of formazan produced is directly proportional to the number of live cells^13^.

## Materials and Methods

A total of 126 isolates of *S. aureus* were collected from tertiary healthcare center in Amravati region (Maharashtra, India) from March 2013 to October 2015 and were confirmed by standard tests like catalase, slide and tube coagulase and growth on mannitol salt agar. The isolates were obtained from mostly the pus and the blood stream infections with due consent from the subjects. No two strains were from the same sample. Standard ATTC strains of MRSA 33591 and MSSA 29213 were also used. All the methods except the newly developed MTT method were carried out as per the Standard Operative Procedures of CLSI following GMT. The methods were carried out according to the guidelines of Indian Council for Medical Research with biosafety level II and were approved by the Institutional Ethical Committee of Sant Gadge Baba Amravati University, Amravati.

### Oxacillin Disk Diffusion Method

Disk diffusion method was performed on Mueller Hinton agar plate with 4% NaCl. The plates were inoculated by 0.5 McFarland standard inoculum by spreading with sterile cotton swab. Then oxacillin disk of concentration 1 μg was placed on plate and were incubated at 35 °C for 24 h. After incubation zone around the disk was measured. Zone diameter of ≥13 mm, 11–12 mm and ≤10 mm was considered oxacillin susceptible, intermediate and resistant respectively^9^. Test was carried out in triplicate for each strain.

### Oxacillin Agar Dilution Method

*Staphylococcus aureus* were screened for reduced oxacillin susceptibility by agar dilution method. Bacterial suspensions were prepared from overnight cultures on Mueller Hinton agar and their turbidity was adjusted to be equivalent to that of 0.5 McFarland standards. This suspension was inoculated on to Mueller Hinton agar containing serial dilutions of oxacillin. Inoculation of isolates along with control was performed without any antibiotic and was incubated at 35 °C for 24 h^9^. After incubation inhibited growth on particular concentration indicated the MIC for that strain. Test was carried out in triplicate for each strain.

### Oxacillin screening agar test

Oxacillin screening agar test was performed on Mueller-Hinton agar (Hi Media) with 6 μg/ml oxacillin concentration using guidelines for detection of MRSA. Plates were inoculated with 10 μL of 0.5 McFarland bacterial suspensions and incubated for 24 h. Test was carried out in triplicate for each strain.

### Easy MIC test

Easy MIC test was performed on all isolates according to the manufacturer’s instruction (Hi Media, Mumbai). Briefly a bacterial suspension of a 0.5 McFarland standard inoculum in sterile broth was spread on Mueller- Hinton agar plate. Then Oxacillin strip was placed onto the plate with bacterial suspension and the plate was incubated at 35 °C for 24 h. After incubation visual break points were observed around strips. Isolates were categorized as susceptible or resistant to oxacillin according to the break points published by CLSI. Test was carried out in triplicate for each strain.

### MTT based method

**Figure 1:**
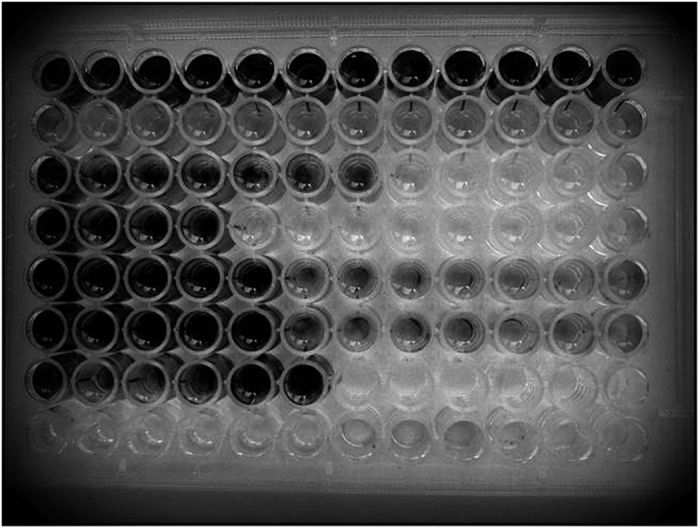
Photoplate showing MIC breakpoints with no colour formation.

A 96 well Tissue culture plate was taken, to which was added the 100 μl of 0.5 McFarland bacterial culture in each well, then 100 μl of sterile broth and different oxacillin concentrations (2, 4, 6, 8, 10, 12, 14, 16, 32, 48, 64, 128, 256, 512 μg/ml) were added to each well, plate was incubated at 37 °C for 6 h. After 6 h incubation, 20 μl of solution of MTT (Hi Media Ltd, Mumbai) (5 mg/ml) in phosphate buffer was added in each well and again the plate was incubated for 1 h. The blue color formation indicated the presence of viable bacteria (Fig. 1) and MIC were determined by visual breakpoints at respective drug concentration. Test was carried out for more than five times for each strain.

### Amplification of *mec*A gene

Four to five colonies of an overnight bacterial subculture were suspended in100 μl nuclease free water. Cells were lysed by heating the suspension at 100 °C for 1 min. The lysate was used as template DNA for amplification. PCR reaction mixture consisted of 2× PCR Master mix (Fermentas Life Sciences, USA) (*Taq* DNA polymerase (recombinant) 0.05 U/μl, Magnesium Chloride 4 mM, dNTPs mix 0.4 mM each), 0.4 μM of each forward (5′-TGAGTTCTGCAGTACCGGAT-3′) and reverse (5′-ATGATTATGGCTCAGGTACTGCTATCCACC-3′) primers described by Weller^14^ were added to PCR master mix. Bacterial lysate (3 μl) was added to PCR Master Mix as a source of template DNA. Final reaction mixture was adjusted to 25 μl. The amplification reaction was carried out for 3 min at 95 °C for initial denaturation. Cycles were done at 1 min for denaturation at 94 °C, 55 °C for 1 min. for annealing of primers, 72 °C for 2 min. for extension and 72 °C for 5 min for final extension. The amplification reaction was repeated for 34 cycles. Amplified product was fractionated on 1.5% agarose gel along with 100 bp ladder (Fermentas US).

## Results

Total 126 strains of *S. aureus* were tested by different methods recommended by CLSI along with the MTT based rapid colorimetric method. In MTT method formazan production could be seen by naked eye and is directly proportional to the bacterial concentration and amount of drug present in single aliquot. The bacterial suspension with turbidity corresponding to turbidity 0.5 McFarland gave the visible formazan formation after 6 h in resistant strain at particular MIC (photo plate 1). All 126 strains of *S. aureus* and their MIC were determined by this method with respect to control and blank. The results for MIC obtained by MTT based method matched to conventional method recommended by CLSI. Table 1 shows the relative concordance between MIC’s obtained by MTT based method, agar dilution method and Easy MIC method along with agreement with reference method. Oxacillin MIC for 16 + 7 (total 23) *S. aureus* isolates was found to be more and for 12 + 3 (total 15) isolates was less than 2 μg/ml (just one reading difference) by the MTT method as compared to the reference method i.e. Easy MIC method, indicating minor errors. For the remaining 88 isolates oxacillin MIC was correctly determined by the test method indicating 98.14% agreement with the reference method. Easy MIC was used as the reference method because as per Table 2, the test was found to be more reliable than the other tests. All oxacillin resistant strains were correctly identified by their higher MIC’s on the basis of resistance criterion given by CLSI guidelines for Antimicrobial Susceptibility testing and Microbial drug resistance. Among 126 *S. aureus* strains, 98 were *mecA* positive and 28 were *mecA* negative (77.78% prevalence). On PCR amplification of *mecA* gene of 785 bp product was obtained. This was considered as gold standard for comparison.

Evaluation of different methods for determination of oxacillin resistance in *S. aureus* with their sensitivity, specificity, likelihood ratios and positive and negative predictive values is described in Table 2. All the values were calculated using a clinical calculator/statistical software for evaluation of diagnostic test. There are 98 MRSA and 28 MSSA as per the gold standard method. MTT method identified all the MRSA except one with sensitivity of 98.98% and identified all MSSA except one with specificity of 96.43%. Of 98 true MRSA isolates, Oxacillin disc diffusion test falsely read 6 as MSSA (false negative) and of 28 MSSA isolates it could only recognize 7 as true negative showing only 25% specificity. While Oxacillin screen agar showed 97.96% sensitivity and 75% specificity. Easy MIC and Agar dilution showed almost similar results with 97.96% and 96.94% sensitivity and 89.29% and 85.71% specificity respectively.

Comparison of different methods also indicated that media requirement per test was very less (300 μl), which defined the higher media requirements for other methods. Agar dilution method was more complex than Easy MIC and MTT based method simpler than Easy MIC and agar dilution method. All the methods required more incubation time. But in case of MTT based method it was only 7 h (Table 3).

## Discussion

There were 98 MRSA and 28 MSSA as tested by PCR along with the standard strains as mentioned in materials and methods. The MTT based method showed similarity with the reference method. All MRSA except one were correctly identified with naked eye, suggesting that visual reading could be sufficient to interpret the results. Technically the test was easy to perform with very rapid results. Early diagnosis of infection with multi-drug resistance of *S. aureus* is important for individual case management as well as for control of spread of MRSA in hospital and non-hospital areas. In this study we developed an affordable and reliable colorimetric method for a rapid detection of oxacillin resistance in *S. aureus* isolates. This shortens the time required for susceptibility reporting by 17 h as compared to the standard method. The maximum period required to complete all the investigations with the MTT based colorimetric method for the strains analyzed in this study was 7 h. A colorimetric assay using MTT was first introduced by Mossman as a quantitative measure of mammalian cell survival and proliferation^15^.

Strains that possess *mecA* classic resistance are either heterogeneous or homogeneous in their expression of resistance. Presence of resistance in *S. aureus* on oxacillin screen agar plate generally means that they are *mecA* positive. Occasionally, however, hetero resistant *mecA* positive strain is not detected due to low expression of resistance. Oxacillin screen generally does not detect borderline resistant strain^4^.

As already reported, the oxacillin disc diffusion test was the least reliable for detection of resistance to oxacillin in *S. aureus*. The high false positivity of oxacillin disc diffusion method in this study could be due to hyper production of β- lactamases which may lead to phenotypic expression of oxacillin resistance, while they appear oxacillin resistant but do not possess the usual genetic mechanism for such resistance. Probably these isolates under antibiotic pressure may evolve into fully resistant isolates subsequently^16^. Or these isolates might have been positive for blaZ gene^6^.

Oxacillin screen agar is recognized as sensitive and specific test^10^,^12^,^16^,^17^,^18^,^19^,^20^ but in our study the specificity was only 75% as it falsely recognized 7 isolates as MSSA.

Since oxacillin susceptibility result may be obtained in just 7 hours by MTT based colorimetric method than the time required for recommended CLSI methods and result were in concordance with standard method (98.31% agreement) and PCR for *mec*A, we suggest that the MTT based colorimetric method may be acceptable and cheap allowing for their routine use in facility constrained laboratories without access to commercial methodology. The validity of this method requires conformation by testing of more oxacillin resistant strains by more laboratories.

## Additional Information

**How to cite this article**: Ghanwate, N. *et al.* Colorimetric method for rapid detection of Oxacillin resistance in *Staphylococcus aureus* and its comparison with PCR for *mec* A gene. *Sci. Rep.* **6**, 23013; doi: 10.1038/srep23013 (2016).

## Figures and Tables

**Table 1:** Comparison of oxacillin MICs by Agar dilution, Easy MIC Test and MTT based method along with errors and agreement with the reference method.

No. of strains with similar readings. (Total-126)	Easy MIC Test* (μg/ml)	Agar dilution Method (μg/ml)	MTT based Method (μg/ml)	Errors		Percent agreement
				Minor	Major	
21	4	4	4	00	00	100
29	6	6	6	00	00	100
03	4	6	4	00	00	100
16	8	8	10	>2(0.25)	00	95.83
31	12	12	12	00	00	100
07	12	16	14	>2(0.25)	00	95.83
12	16	12	14	>2(0.25)	00	95.83
03	6	6	4	>2(0.25)	00	95.83
04	6	4	6	00	00	100
				1.85%	00%	98.14%

^*^Reference method.

**Table 2:** Evaluation of different methods for determination of oxacillin resistance in *S. aureus* with their sensitivity and specificity.

Test	Staphylococcus aureus (126)				Sensitivity %	Specificity %	Positive Likelihood Ratio	Negative Likelihood Ratio	PPV# %	NPV+ %
	*PCR for mecA									
	mecA +ve (98) (MRSA)		mecA −ve (28) (MSSA)							
	True positive	False negative	True negative	False positive						
Oxacillin Screen Agar	96	2	21	7	97.96	75	3.92	0.03	93.20	91.30
**MTT based method**	**97**	**1**	**27**	**1**	**98.98**	**96.43**	**27.71**	**0.01**	**98.98**	**96.43**
Easy MIC test	96	2	25	3	97.96	89.29	9.14	0.02	96.97	92.59
Oxacillin Disc Diffusion (1 ug)	92	6	7	21	93.88	25.00	1.25	0.24	81.42	53.85
Agar Dilution	95	3	24	4	96.94	85.71	6.79	0.04	95.96	88.89

^*^Reference method.

^#^Positive Predictive Value.

^+^Negative Predictive Value.

**Table 3:** Technical Comparison between different methods and MTT based method.

Method	Media requirement per test	Time required	MIC determination	Complexity
**Agar dilution Method**^9^	300 ml	24 h	Yes	More tedious with higher media requirement
**Easy MIC Test**^9^	20 ml	24 h	Yes	Required Easy MIC strips which increased the cost
**MTT based Method**	**300 μl**	**7 h**	Yes	**Very less media required, rapid and simple**
**Oxacillin Screen Agar**^9^	20 ml	24 h	No	Cannot detect borderline resistant strains
**Oxacillin Disc Diffusion**^9^	20 ml	24 h	No	Gives false positive results
**PCR for** ***mec*****A**^14^	–	12–16 h	No	Set up not available in all laboratories
